# Comparison of tobacco-specific nitrosamine levels in smokeless tobacco products: High levels in products from Bangladesh

**DOI:** 10.1371/journal.pone.0233111

**Published:** 2020-05-26

**Authors:** Shamema Nasrin, Gang Chen, Christy J. W. Watson, Philip Lazarus

**Affiliations:** Department of Pharmaceutical Sciences, College of Pharmacy and Pharmaceutical Sciences, Washington State University, Spokane, Washington, United States of America; University of Calfornia San Francisco, UNITED STATES

## Abstract

Bangladesh exhibits the second highest rate of smokeless tobacco (SLT) product usage in the world, and this has been associated with the high upper aerodigestive tract cancer incidence in this country. The goal of the present study was to examine the levels of the highly carcinogenic tobacco-specific nitrosamines (TSNAs) in Bangladeshi SLT products and compare these levels to that observed in SLT brands from southeast Asia and the USA. The levels of TSNAs and nicotine were determined by LC-MS/MS in twenty-eight SLT brands and several tobacco additives from Bangladesh, as well as several SLT brands from India, Pakistan and the USA. The levels of N-nitrosonornicotine (NNN), 4-(methylnitrosamino)-1-(3-pyridyl)-1-butanone (NNK), N-nitrosoanatabine (NAT) and N-nitrosoanabasine (NAB) in Bangladeshi SLT brands ranged from 1.1–59, 0.15–34, 0.79–45, and 0.037–13 μg/g SLT powder, respectively. The mean levels of the highly carcinogenic TSNAs (NNN+NNK) were 7.4-, 2.4-, and 63-fold higher in Bangladeshi SLT products as compared to SLT brands from the USA, India and Pakistan, respectively; these trends were also observed for NAT and NAB. Similar mean levels of nicotine were observed in the Bangladeshi brands (31 mg/g powder) versus brands from the USA (25 mg/g powder) and India (20 mg/g powder); they were 3-fold higher than brands from Pakistan (10 mg/g powder). Gul SLT brands exhibited the highest pH and the highest levels of unprotonated nicotine. The high levels of TSNAs in Bangladeshi SLT brands may be an important factor contributing to the high rates of upper aerodigestive tract cancer in Bangladesh.

## Introduction

Tobacco use has long been recognized as a contributing factor for cancers of the aerodigestive tract [1–4] and is also strongly associated with many other diseases including chronic obstructive pulmonary disease and cardiovascular disease [5–8]. Tobacco is consumed in several forms in developed and developing countries worldwide as both a smoked or smokeless product. Smoked tobacco is frequently marketed as factory-made cigarettes and cigars, or as loose tobacco smoked from a pipe or hand-rolled cigarettes. Smokeless tobacco (SLT) also comes in many forms including snus, chew, toombak (fermented tobacco), zarda (flavored tobacco flakes), gul (powdered tobacco), sada pata (tobacco leaf), and khaini (flavored tobacco flakes mixed with slaked lime). Smokeless tobacco is placed in contact with mucus membranes, either placed inside the mouth between the cheek and gums or chewed, and has long been associated with squamous cell carcinoma in various head and neck (H&N) tissues [9–12].

SLT contains a variety of weak carcinogens including volatile aldehydes such as formaldehyde, acetaldehyde and crotonaldhyde [13]. Also present are the volatile nitrosamines, N-nitrosodimethyl-amine and N-nitrosopyrrolidine, polonium [14] and low levels of some polycyclic aromatic hydrocarbons [PAHs; [15]]. The tobacco-specific nitrosamines (TSNA) are the most abundant carcinogens in SLT and are produced by nitrosation of tobacco alkaloids during the tobacco curing and fermentation process [16, 17]. They are considered to be major contributors in the induction of cancers of the respiratory and upper aerodigestive tracts [18].The two main carcinogenic compounds in this group are 4-(methylnitrosamino)-1-(3-pyridyl)-1-butanone (NNK) and *N-*nitrosonornicotine [NNN; [19]]. In humans, NNK is rapidly metabolized by carbonyl reduction to its equally potent carcinogenic metabolite, 4-(methylnitrosamino-1-(3-pyridyl)-1-butanol [NNAL; [20]]. Both NNK and NNAL are metabolized into intermediates which bind to human lung DNA, transform human epithelium *in vitro*, and induce non-tumorigenic human bronchial epithelial cells to become neoplastically transformed in nude mice after subcutaneous transplantation [21]. Tissue-specific carcinogenicity is observed in rodent tissues for NNN and NNK [17]. NNK induces primarily lung adenocarcinomas in rodents independent of the route of administration [22] and is therefore strongly implicated as a human lung carcinogen [23]. (*S*)-NNN, the most abundant enantiomer of NNN in tobacco, is highly carcinogenic in rats, forming both oral and esophageal tumors [24–26]. In addition, tumor induction in the oral cavity by tobacco constituents was observed after co-application of NNN and NNK to the oral cavity of F344 rats at doses similar to that of a chronic snuff user (10 g/day) after 40 y of consumption of smokeless tobacco [27, 28]. These data provide strong support for the role of NNN and NNK as major causative factors in tissue-specific tobacco-related carcinogenesis. Two other TSNAs, *N*'-nitrosoanabasine (NAB) and N'-nitrosoanatabine (NAT), are also present in SLT tobacco products at comparable levels to NNN and NNK; however, they are either not or only weakly carcinogenic in rodent models [29].

Aerodigestive tract cancer rates in Bangladesh are among the highest in the world [30]. These high rates may be due to the high prevalence of smoking and/or SLT consumption in this population. In Bangladesh, 43% of the adult population (41.3 million people) use some form of tobacco, of which 45% are male smokers, 1.5% are female smokers, 26% are male SLT users and 28% are female SLT users [31–33]. The high rate of tobacco use, as well as sex differences in the use of smoked versus smokeless tobacco products, can be attributed to societal expectations that disapprove of women smoking, but often encourage women to use SLT products.

There are different types of SLT found in Bangladesh and there is variation in the type of SLT use and in consumption patterns across the Bangladeshi population. The two major brand types of Bangladeshi SLTs are the zarda and gul brands. Zarda is a processed tobacco flake usually boiled, baked, or roasted with or without slaked lime and spices, sweeteners and different types of flavors, especially menthol and camphor, herbs, fragrances, saffron and silver flakes. These products are usually chewed with betel leaf, mineral lime and/or areca nut [32]. These SLTs are considered a confectionary item, available in a variety of flavors with accompanying condiments added for flavor variety, such as coconut, fennel seed, sugar, clove, cinnamon, nuts, and slacked lime. Zarda can include three different forms of dry flakes known as ‘shukna zarda’, brands with wet or moist flakes soaked with menthol/camphor known as ‘bhija patti zarda’, and flakes flavored with saffron called ‘*zafrani zarda’*. Similar forms of SLT in the U.S. are known as moist snuff.

Gul is usually a powdered mixture of tobacco and slaked lime, sometimes containing areca nut and other ingredients like catechu (Kattha), peppermint and cardamom. These products are applied on teeth and gums as dentifrice, placed between the cheek and gums, or placed in the nasal cavity as snuff. Another SLT product commonly used in Bangladesh is sada pata, which is comprised of plain dried tobacco leaves, usually without any processing, and is often chewed along with zarda, betel leaf and areca nut. While not classified as a typical SLT product, Pan masala is a sweet mixture of betel nut, sugar chunks, coconut, fennel seed, and specialty regional flavors, sold as a non-tobacco product and often consumed by children in the same fashion and can itself be carcinogenic [34]. It is sold with many of the same flavors as SLT products, are often used in combination with tobacco products in younger population [35, 36].

There is a lack of available data on the chemical constituents of the many different forms of SLT products available in Bangladesh, and no previous studies have examined the levels of TSNA in Bangladeshi SLT products. The goal of the present study was to directly quantify the levels of TSNAs in popular SLT brands from Bangladesh. Additionally, previous studies have suggested that increased levels of TSNAs may be directly related to tobacco moisture content, as moisture may promote microbial growth which contributes to increased nitrosation, an important step in the formation of TSNAs during processing and storage [37]. Also, tobacco acidity levels may be a determinant of the fraction of unprotonated nicotine in SLT products since the uncharged form is most readily absorbed during oral tobacco use [38, 39]. Therefore, the pH, moisture content and nicotine content were also measured in these Bangladeshi products to better characterize their chemical composition.

## Materials and methods

### Tobacco samples

Thirty-four brands of SLT widely used in different regions of Bangladesh were chosen for this study, including several popular brands of zarda, gul and sada pata [32]. All SLT products were purchased and collected from retail stores in the Bangladeshi cities of Dhaka, Chittagong, Rajshahi and Khulna in November and December of 2016. Included among these SLT products were 22 popular brands corresponding to ‘zarda’. Four brands of powdered snuff commonly known as ‘gul’ and six brands of non-tobacco additives (three Pan masala brands and 3 types of betel nut) were also collected. Six common U.S. brands of snuff were purchased from local tobacco shops in Spokane, WA in December 2016, while six Indian and two Pakistani brands were purchased from Mumbai, India and Karachi, Pakistan in March and April, 2017. All products were shipped to the laboratory at Washington State University (Spokane, WA) within 25 days after their purchase and were immediately stored at 4°C until the time of analysis.

Two CORESTA (Cooperation Centre for Scientific Research Relative to Tobacco) Reference Products (CRP) were donated by the Smokeless Tobacco Reference Products Program (North Carolina State University, Raleigh, NC), and used as controls to verify the quantification method for individual TSNAs and nicotine: CRP-1.1, Swedish style snus pouch; and CRP-2.1, American-style loose, moist snuff. These certified reference products (CRP) are purposely manufactured for laboratory analysis by the Tobacco and Volatiles Branch of the Division of Laboratory Sciences at the Centers for Disease Control and Prevention ([CDC] Atlanta, Georgia, USA).

### Reagents

Nicotine, NNK, NNN, NAT and NAB standards and their corresponding d4-labeled internal standards were purchased from Toronto Research Chemicals (Toronto, Canada). UPLC-MS grade solvents including methanol and ammonium acetate were purchased from Fisher Scientific. Ultra-pure water (MilliQ Q-POD) was used for all analysis.

### Analytical methods

**Fig 1:**
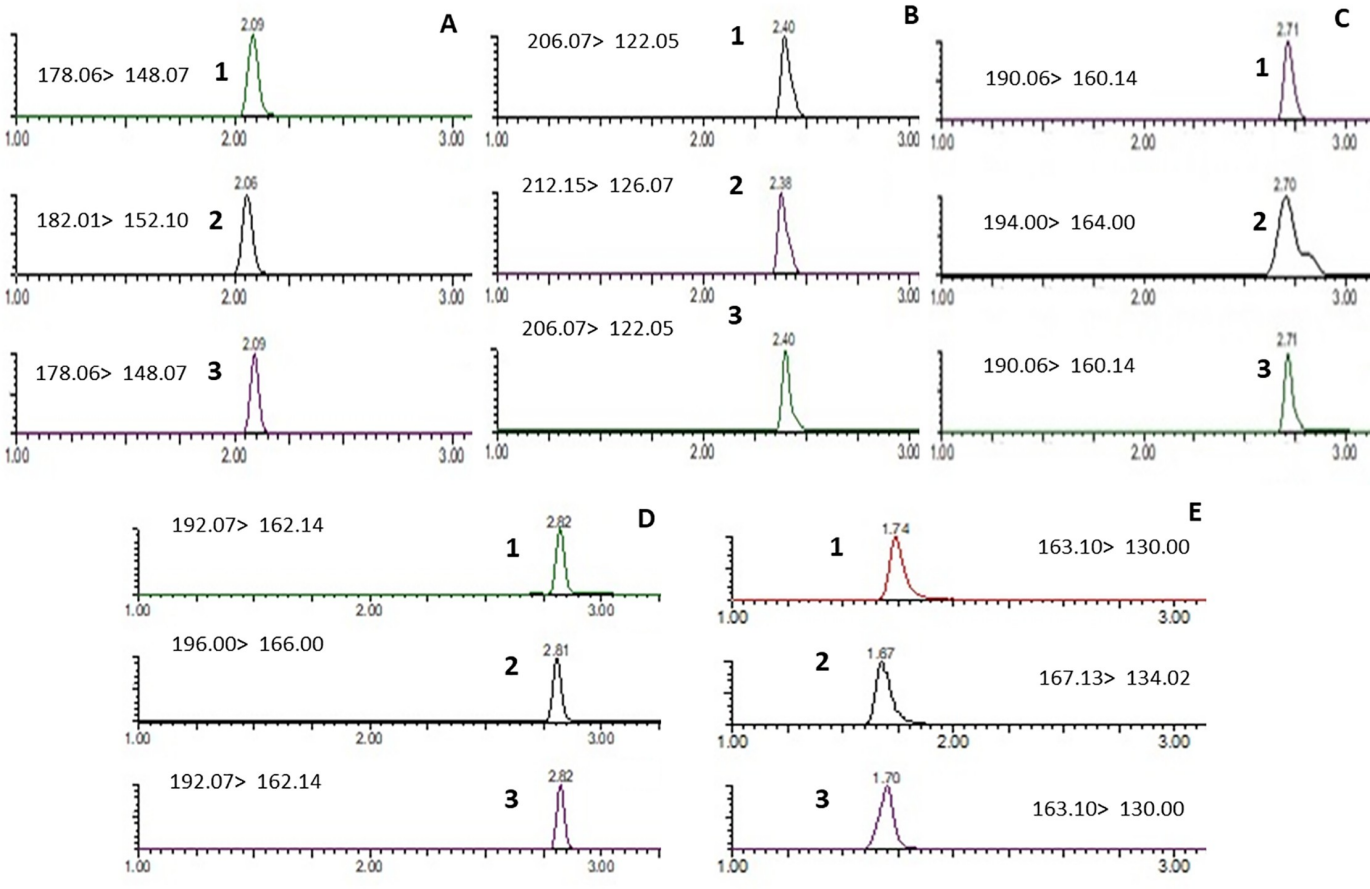
Representative LC-MS/MS chromatograms showing the separation and detection of individual TSNAs and nicotine. **Panel A**: Peak 1, NNN standard; peak 2, NNN-d4 standard; peak 3, NNN extracted from SLT sample (Akiz zarda). **Panel B**: Peak 1, NNK standard; peak 2, NNK-d4 standard; peak 3, NNK extracted from SLT sample (Akiz zarda). **Panel C**: Peak 1, NAT standard; peak 2, NAT-d4 standard; peak 3, NAT extracted from SLT sample (Akiz zarda). **Panel D**: Peak 1, NAB standard; peak 2, NAB-d4 standard; peak 3, NAB extracted from SLT sample (Akiz zarda). **Panel E**: Peak 1, nicotine standard; peak 2, nicotine-d4 standard; peak 3, nicotine extracted from SLT sample (Akiz zarda). Mass transition values for each compound are shown within their respective panels.

Immediately upon the opening of an SLT product, samples were ground to a fine powder using a mortar and pestle and stored at 4ºC for subsequent nicotine and TSNA content analysis. The extraction and quantification of nicotine and TSNAs was performed by a modification of the method previously used and recommended by CORESTA. For the extraction of TSNAs and nicotine from SLT products, approximately 100 mg (for TSNA analysis) or 20 mg (for nicotine analysis) of ground SLT powder were transferred into a 50 mL conical flask containing internal standard (for TSNA quantification: 10 ppm each of NNN-d4, NNK-d4, NAT-d4 and NAB-d4; for nicotine quantification: 100 ppm of nicotine-d4) and 15–20 mL 100 mM ammonium acetate. After 40 min of shaking at 180 rpm in an orbital shaker at room temperature, each sample was filtered using a 0.2 μm syringe filter. Analysis was performed using an Acquity H class ultra-pressure liquid chromatograph (UPLC; Waters) equipped with a BEH C18 (2.1 X 50 mm, 1.7 μm) UPLC column at 45°C. Analytes were separated using a gradient elution at 0.4 mL/min under the following conditions: 0.5 min with 90% buffer A (5 mM ammonium acetate) and 10% buffer B (100% MeOH), followed by a linear gradient for 3.0 min to 70% buffer B and a subsequent linear gradient for 1.0 min to 90% buffer B. Flow continued for 1 min in 95% buffer B before re-equilibrium in 10% buffer B for 2 min. Analytes were detected using a Waters Xevo TQD tandem mass spectrometer equipped with a Zspray electrospray ionization interface operated in the positive ion mode, with the capillary voltage at 0.6 kV. Nitrogen was used as both the cone and desolvation gas at 50 and 800 L/h, respectively. Ultrapure argon was used for collision-induced dissociation. The desolvation temperature was 500°C. For the detection of TSNAs and nicotine, the mass spectrometer was operated in multiple reaction-monitoring mode (MRM). The ion-related parameters monitored for each transitions are provided in Fig 1. The minimum levels of detection (LOD) for nicotine and each of the TSNAs was 1 and 5 ng/g SLT powder, respectively.

Previous studies have suggested that increased levels of TSNAs may be directly related to tobacco moisture content, as moisture may promote microbial growth which contributes to increased nitrosation, an important step in the formation of TSNAs during processing and storage [37]. Studies have also suggested that tobacco acidity levels may be a determinant of the fraction of unprotonated nicotine in SLT products since the uncharged form is most readily absorbed during oral tobacco use [38, 39]. The moisture content and pH were immediately determined after preparing each SLT powder. The analytical methods used for determination of moisture content (oven volatile) and pH in SLTs followed previously reported method. Briefly, 2 g of ground SLT samples were mixed with 20 ml of water, and after 15 min of shaking, the pH of the slurry was measured. For moisture content, 2 g SLT powder was weighed, dried at 100°C for 3 h, and weighed again; the difference in weight was attributed to water content within each SLT product.

### Data analysis

Data were analyzed using the Masslynx 3.5 software provided by Waters. All chromatogram peaks were reviewed, and integration manually corrected when necessary. Calibration curves were analyzed using linear regression with 1/x weighting. All standard and sample concentrations were determined using internal standard peak area versus analyte peak area. The Welch’s t-test was used to compare, (i) TSNA, and (ii) nicotine levels in SLT products from different countries. Unprotonated nicotine was calculated as a percentage of total nicotine using the Henderson–Hasselbalch equation [40]. The F-test was used to compare variance within different brand types, and Pearson coefficient analysis was used to assess potential correlations between, (i) TSNA and nicotine levels, and (ii) TSNA or nicotine content and pH, within the Bangladeshi brands. All statistical analysis was performed using Graphpad prism 6 software.

## Results

The Bangladeshi SLT brands that were examined in this study included zarda, gul, and sada pata brands. Bangladeshi SLT products were first extracted and then analyzed by LC-MS/MS. The representative LC-MS/MS chromatograms shown in Fig 1 demonstrate the effective separation of individual TSNAs as well as nicotine in a representative Bangladeshi SLT product (Akiz zarda). These peaks exhibited the same retention times as their corresponding purchased standards and similar retention times as compared to their corresponding purchased internal standards.

**Fig 2:**
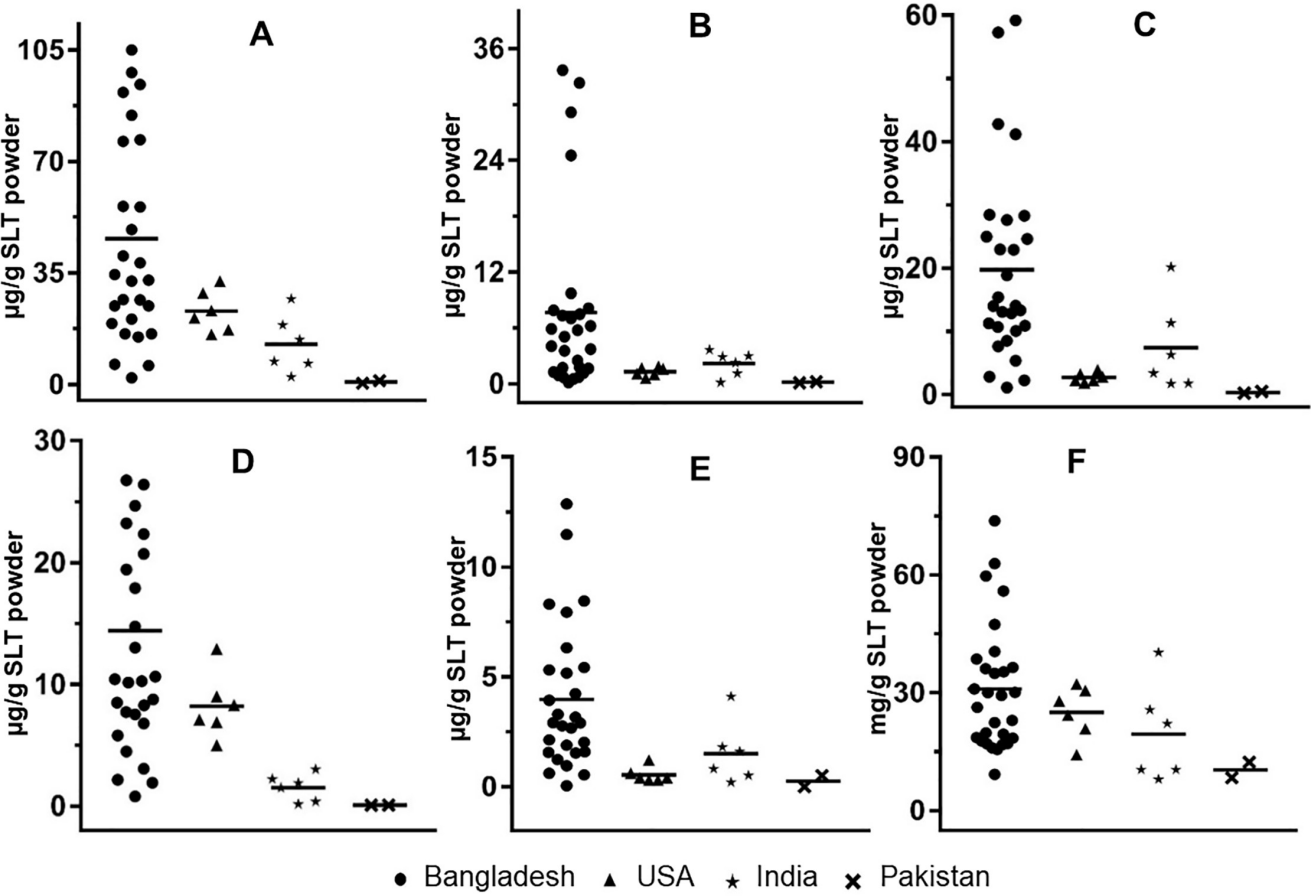
Box plot showing the mean values and distribution of TSNAs and nicotine in Bangladesh, USA, India and Pakistan SLT brands. Panel A, total TSNA; Panel B, NNK; Panel C, NNN; Panel D, NAT; Panel E, NAB; Panel F, nicotine.

The TSNA values obtained in the present study for the reference tobacco products were consistent with the certified reference values (TSNA range for CRP 1.1 were NNN: 0.13–0.42 μg/g SLT powder, NNK: 0.032–0.078 μg/g SLT powder, NAT: 0.083–0.20 μg/g SLT powder, NAB: 0.014–0.004 μg/g SLT powder, nicotine: 4.9–8.2 mg/g SLT powder; CRP 2.1 were NNN: 2.9–7.3 μg/g SLT powder, NNK: 1.7–2.4 μg/g SLT powder, NAT: 3.8–4.9 μg/g SLT powder and NAB: 0.22–0.56 μg/g SLT powder, nicotine: 8.0–13 mg/g SLT powder; Table 1) provided by the Cooperation Centre for Scientific Research Relative to Tobacco (CORESTA). The levels of total TSNA vary widely in the different Bangladeshi SLT brands (Fig 2A), with total TSNA concentrations ranging from 2.1 to 114 μg TSNA/g SLT powder (Table 1).

**Table 1:** TSNA, nicotine, pH and moisture content in Bangladeshi and reference tobacco products.

SLT brand	TSNA^a^						nicotine^b^			
	NNN	NNK	NAT	NAB	total TSNA	NNN+NNK	total	% unprotonated^c^	pH	% moisture
**Flavored/ non-flavored tobacco flakes (Zarda)**										
Shohag	59	7.5	23	7.9	98	67	30	24	7.4	23
Nurani C	57	6.2	22	8.3	94	63	74	48	7.9	15
Momo	43	8.1	21	13	84	51	23	57	8.0	26
Akiz	41	5.8	18	11	76	47	20	3.0	6.4	28
Chadpuri shah	28	32	45	8.5	114	61	17	8.9	6.9	25
Nurani	28	34	27	2.9	92	62	18	17	7.2	22
Hakimpuri	28	29	42	6.3	105	57	36	8.2	6.9	29
Zakir	25	4.1	15	5.2	49	29	40	1.3	6.0	31
Alo	23	5.1	25	3.2	56	28	22	52	7.9	23
Halimpuri	23	25	26	2.9	77	47	30	44	7.8	28
Gurudev	15	1.6	6.8	2.7	26	17	17	2.0	6.2	24
Dulals	14	5.9	11	2.1	33	20	39	0.46	5.6	27
Bou	13	1.7	7.7	1.9	25	15	36	1.5	6.1	28
Saudia	13	7.4	8.8	3.3	32	20	17	43	7.8	18
Noman	11	1.8	4.5	1.6	19	13	20	3.1	6.4	25
Babul	11	3.6	10	2.0	27	14	16	10	7.0	23
Read leaf	11	2.5	7.5	3.9	25	13	35	5.4	6.7	28
Tofan	10	1.2	3.1	0.53	15	11	18	0.38	5.5	25
Shova	8.5	0.73	8.5	2.8	20	9.2	56	2.7	6.3	31
Ezma	7.6	0.94	5.8	1.5	16	8.6	16	5.4	6.7	25
Murubbi baba	5.3	1.3	8.3	0.94	16	6.6	35	1.3	6.0	27
Aadi bhija	2.8	0.72	2.1	0.61	6.3	3.5	26	4.2	6.5	40
**Powdered tobacco/snuff (Gul)**										
Bogh	25	9.7	19	1.6	56	35	19	99	9.8	12
Mostafa	19	3.7	10	5.3	38	23	60	96	9.3	14
Bidut	14	7.9	13	5.4	40	22	63	96	9.3	14
Shahi eagle	13	7.0	10	4.2	35	20	47	96	9.3	17
**Plain tobacco leafs (Sada pata)**										
Plain non-brand	2.2	0.55	1.9	1.2	5.9	2.7	9.2	14	7.1	20
Shahzadi	1.1	0.15	0.79	0.037	2.1	1.2	29	9.1	6.9	25
**Tobacco additives**										
**Pan masala**										
Panparag	0.69	0.14	0.39	0.020	1.2	0.82	25	0.55	5.6	22
Shahi Deluxe	0.56	0.18	0.53	0.12	1.4	0.74	0.0017	NM^d^	NM	NM
Shahi	<LOD^e^	0.009	<LOD	<LOD	0.0093	0.009	1.6	NM	NM	NM
**Betel nut**										
Dried	ND^f^	ND	ND	ND	ND	ND	ND	NM	NM	NM
Raw	ND	ND	ND	ND	ND	ND	ND	NM	NM	NM
Processed	0.093	ND	0.12	0.025	0.24	0.093	0.49	NM	NM	NM
**Reference product (CORESTA)**										
Reference 1.1	0.40	0.070	0.11	0.0053	0.59	0.47	7.6	56	8.0	44
Reference 2.1	6.0	2.7	3.6	0.19	13	8.7	9.8	18	7.2	50

^**a**^ Each reported value is the mean of duplicate analyses for the individual TSNAs, expressed as μg/g SLT powder

^b^ Mean of triplicate analysis for nicotine, expressed as mg/g SLT powder

^c^ Percentage of unprotonated nicotine was calculated using the Henderson–Hasselbalch equation [40]

^d^ NM, not measured

^e^ <LOD, below the limit of detection

^f^ ND, not detecte

The levels of total TSNA in the 22 zarda and 4 gul brands ranged from 6.3–114 μg/g SLT powder and 35–56 μg/g SLT powder, respectively. There was significantly (p = 0.049) more variability in TSNA content in the zarda brands as compared to the gul brands. By contrast, TSNA values were generally lower in SLT brands from the USA, India and Pakistan, ranging from 4.9–9.8, 2.3–27, and 0.38–1.2 μg/g SLT powder, respectively (Table 2). The mean TSNA content in the Bangladeshi SLT products (including the zarda, gul and sada pata brands) was 46 μg/g SLT powder. This was significantly higher than the SLT brands examined from the USA (*p*< 0.001), India (*p*< 0.001) or Pakistan (*p*< 0.001), where the mean total TSNA content was 7.2 μg/g SLT powder, 13 μg/g SLT powder and 0.81 μg/g SLT powder, respectively (Fig 2). The mean levels of total TSNA were similar in the zarda (51 μg/g SLT powder) vs. gul (42 μg/g SLT powder) brands. While the levels of TSNA in sada pata brands (4.0 μg/g SLT powder; Table 1), were lower than those observed in the gul and zarda brands, this difference was not significant, likely due to the fact that only two sada pata brands were analyzed in this study.

**Table 2:** Levels of TSNA, nicotine, pH and moisture in USA, India and Pakistan SLT brands.

	TSNA^a^										nicotine^b^			% moisture
SLT brand	NNN		NNK	NAT		NAB	total TSNA		NNN+NNK		total	% unprotonated^c^	pH	
**USA brands**														
Skoal long cut	2.2	1.7		3.1		0.61	7.6		3.9	32		56	8.0	55
Copenhagen snuff	3.2	0.65		2.1		0.35	6.3		3.9	31		28	7.5	47
Kodiak premium	1.9	1.6		1.7		0.41	5.6		3.5	24		87	8.7	52
Grizzly long cut	2.8	1.1		4.2		1.2	9.3		3.9	14		17	7.2	52
Grizzly extra long	3.9	1.9		3.7		0.34	9.8		5.8	21		26	7.5	49
Grizzly premium dark	2.2	1.04		1.2		0.47	4.9		3.2	28		57	8.0	50
**Indian brands**														
Baba Zarda	6.3	2.3		1.5		4.1	14		8.6	40		50	7.9	24
Paanparag Zarda	1.8	0.17		0.15		0.2	2.3		1.9	7.9		23	7.4	27
Goa 1000 Zarda	11	3.6		1.9		1.8	19		15	22		9.3	6.9	21
Gopal Zarda	3.4	3.0		0.37		0.53	7.3		6.4	26		46	7.8	15
Tulshi ghutka mix	1.7	1.1		2.2		1.6	6.6		2.8	10		11	7.0	33
Shimla Chap	20	2.9		3.0		0.84	27		23	10		57	8.0	37
**Pakistani brands**														
Manipuri	0.18	0.14		0.073		ND^d^	0.38	0.31		12		11	7.0	18
Naswar	0.46	0.22		0.061		0.51	1.3	0.68		8.4		22	7.4	23

^**a**^ Each reported value is the mean of duplicate analyses for the individual TSNAs, expressed as μg/g SLT powder

^b^ Mean of triplicate analysis for nicotine, expressed as mg/g SLT powder

^c^ Percentage of unprotonated nicotine was calculated using the Henderson–Hasselbalch equation [40]

^d^ ND, not detected.

The levels of NNN and NNK ranged from 2.8 to 59 μg/g SLT powder and 0.15 to 34 μg/g SLT powder, respectively, in the Bangladeshi gul and zarda SLT brands (Table 1). The highest levels of NNN (59 μg/g SLT powder) and NNK (34 μg/g SLT powder) were observed in the Shohag and Nurani zarda brands, respectively. The mean levels of NNN + NNK in Bangladeshi SLT brands (27 μg/g SLT powder) were 7.4-, 2.7-, and 63-fold higher than SLT products from the USA (4.0 μg/g SLT powder; *p <* 0.001), India (9.6 μg/g SLT powder; p = 0.0023) and Pakistan (0.50 μg/g SLT powder; *p <* 0.001; Table 2). Similarly, the levels of NNK were 6.0-, 3.6-, and 44-fold higher in the Bangladeshi brands than that observed in the USA (p = 0.0019), Indian (p = 0.0075), and Pakistani (p = 0.0004) SLT brands, respectively (Fig 2B). The levels of NNN were 7.4-, 2.7-, and 63-fold higher in the Bangladeshi brands than that observed in the USA (*p <* 0.001), Indian (p = 0.0084), and Pakistani (p = 0.0004) SLT brands, respectively (Fig 2C).

Although the highest combined level of NNN + NNK observed in a zarda brand (Shohag zarda, 67 μg/g SLT powder) was almost twice as high as the highest combined level of NNN + NNK in a gul brand (Bogh marka, 35 μg/g SLT powder), the mean combined levels of NNN + NNK were similar in the two Bangladeshi SLT brand groups (zarda = 31 μg/g SLT powder vs. gul = 25 μg/g SLT powder; Table 1). Among the products in which TSNAs were commonly detected, the lowest levels of NNK + NNN were observed in the two sada pata brands. Surprisingly, the tobacco additive Pan masala also contained low levels of TSNAs, with quantifiable levels detected in two of the three brands (Panparag and Shahi Deluxe) and trace levels of NNK observed in the Shahi brand. No TSNAs were observed in either processed, dried or raw betel nut (Table 1).

Similar to that observed for NNN and NNK, the mean levels of NAT (14 μg/g SLT powder; Fig 2D) and NAB (4.0 μg/g SLT powder; Fig 2E) were higher in Bangladeshi brands as compared to SLT products from the USA, India and Pakistani SLT brands. The mean NAT levels in Bangladeshi SLT brands were 5.2-, 9.2-, and 215-fold higher than USA (p = 0.014), India (*p* < 0.0001) or Pakistan (*p* < 0.0001) brands, respectively. Similarly, the levels of NAB were 7.5- (*p* < 0.001), 2.7- (p = 0.009), and 8.0-fold (*p* < 0.0001) higher for Bangladeshi SLT brands than SLT brands from the USA, India and Pakistan, respectively.

The average nicotine content in Bangladeshi SLT products was 31 mg/g SLT powder, with a range of 9.2 to 74 mg/g SLT powder (Fig 2F). The average amount of nicotine in the USA, India and Pakistani brands were 25, 20 and 10 mg nicotine/g SLT powder, respectively (Fig 2), which were 1.2-, 1.6-, and 3-fold lower than that observed for Bangladeshi SLT brands, respectively. The average nicotine levels in zarda, gul and sada pata were 29, 47 and 19 nicotine/g SLT powder, with the highest levels (74 mg/g SLT powder) observed in the Nurani zarda brand. Low levels or no detectable nicotine were detected in two of the three pan masala brands and in brands of betel nut. No correlation between the levels of nicotine and TSNAs were observed in Bangladeshi SLT brands.

The moisture content for Bangladeshi tobacco products ranged from 12 to 40% (Table 1). The mean moisture content for zarda SLT products (26%) was comparable to that observed in gul SLT brands (23%) and was slightly higher than that observed for the sada pata brands (15%). The mean moisture content of the Bangladeshi brands (24%) was similar to that observed for brands from India (26%) and Pakistan (21%) and was approximately 2-fold less than that observed for USA brands (51%).

The pH of Bangladeshi SLT products ranged from 5.6 to 9.8, with the pH of gul products (9.3 to 9.8) higher than that observed for other brands (Table 1). The mean pH for the zarda (mean pH = 6.8) and sada pata (mean pH = 7.0) brands were similar to that observed for brands from the USA (mean pH = 7.6), Indian (mean pH = 7.5) and Pakistani (mean pH = 7.2). The four gul products also exhibited the highest levels of unprotonated nicotine (96–99%; Table 1). Those zarda brands which exhibited a moderate to high pH (7.8–8.0; Nurani C, Momo, Alo, Hakimpuri, and Saudia) also exhibited higher levels of unprotonated nicotine (between 43 and 57%), while brands of lower pH exhibiting lower levels of unprotonated nicotine. This pattern was similar to that observed for SLT brands from the USA, India, and Pakistan, as well as the CORESTA reference products.

## Discussion

Thirty-four Bangladeshi SLT products, including several popular brands of zarda, gul and sada pata, several tobacco-free chewing products (betel nut), and several tobacco flavorings/mouth fresheners (pan masala), were analyzed for levels of TSNAs and nicotine as well as pH and moisture content and compared to that observed in SLT brands from India, Pakistan and the USA. Our study shows that the levels of TSNAs in these products can vary widely, with extraordinarily high levels observed in two different brands of zarda (Chadpuri shah and Hakimpure, with 114 and 105 μg TSNA/g SLT, respectively), which are among the most popular forms of SLT in Bangladesh [31]. These levels are higher than that reported for any tobacco product grown outside Africa, and are approximately 4-5-fold lower than the mean total TSNA content observed in Sudanese toombak, which was shown to contain the highest levels of TSNA ever reported for a tobacco product [13, 41–46].

NNN and NNK levels were higher in most Bangladeshi SLT brands, as compared to SLT brands examined from India, Pakistan and the USA (18 out of 22 brands were higher for NNN, and 16 out of 22 brands were higher for NNK). This included 14 brands of zarda and all 4 brands of gul examined in this study. NAT and NAB level were also higher in Bangladeshi zarda and gul brands, with the levels of NAT higher in 24 of the 26 Bangladeshi brands examined. NAB levels were higher in 10 of the Bangladeshi brands as compared to the highest levels observed in any brand from India, Pakistan or the USA.

The levels of nicotine in Bangladeshi SLT products are comparable to that observed elsewhere [41, 47]. The current study found that the average level of nicotine in Bangladeshi SLT products is 29 mg/g SLT powder, with the highest observed in the Nurani zarda brand at 74 mg/g SLT powder, which is the highest among all tobacco products ever reported globally [13, 41, 42, 45–48]. Interestingly, no correlation was observed between nicotine content and TSNA levels in the Bangladeshi SLT products. In fact, despite higher levels of nicotine, the gul brands exhibited slightly lower levels of TSNAs as compared to zarda SLT products.

Nicotine conversion and nitrate content have been suggested to be important factors that contribute to the accumulation of TSNAs in tobacco products [37]. One possible reason for higher TSNA content in Bangladeshi SLT products is that Bangladeshi SLTs are mainly from *N*. *rustica*, a high nicotine-containing tobacco plant species [41, 47]. The high variability observed in TSNA levels for Bangladeshi SLT products may also be due to differences in the manufacturing process of these SLT products, resulting in greater product variability [32]. In Bangladesh, air-cured leaves are generally stored on the farm before selling to tobacco companies where they are then stemmed and re-dried, followed by 1–2 years of storage in warehouses. They are then processed by air-curing and further drying, with nitrates released from the tobacco leaves. Nitrates are chemically unstable compounds that can form TSNAs by directly reacting with tobacco alkaloids, or via microorganism digestion [32]. Large differences in TSNA formation could be dependent on a variety of factors including time of storage, air quality during storage, and temperature and humidity during storage [49]. In addition, the use of the large amount of nitrogenous fertilizers and chemicals including urea, TSP (Triple super phosphate) and pesticides may also contribute to higher TSNA formation [50].

Previous studies showed that increasing the alkalinity of SLT promotes the absorption of nicotine and increases its physiological effects [51]. The majority of the Bangladeshi SLT products examined in this study were moderately acidic in the pH range of 5.5–6.9, which is similar to that observed in SLT brands examined from other countries. Interestingly, all four Bangladeshi gul SLT products exhibited very high pH values (mean = 9.4), and these same four products also exhibited the highest levels of unprotonated nicotine. The zarda brands which exhibited a moderate to high pH (Nurani C, Momo, Alo, Hakimpuri, and Saudia) also exhibited higher levels of unprotonated nicotine while brands of lower pH exhibiting lower levels of unprotonated nicotine. This pattern was similar to that observed for SLT brands from the USA, India, and Pakistan, as well as the CORESTA reference products. These data suggest variability in the potential for increased absorption of nicotine due to pH differences in the different SLT products from Bangladesh and that gul SLTs may contain the highest addictive potential of all Bangladeshi SLT products due to high pH-driven increases in the absorption of nicotine.

All of the tobacco additives in Bangladesh (panparag, sweet pan masala and shahi deluxe pan masala) are labeled as non-tobacco products, however the present study demonstrated that they all contain low levels of nicotine and TSNA compounds. Raw and dried betel nut did not contain detectable levels of nicotine or TSNAs, which is expected given that betel nut is also a non-tobacco product. It was interesting that trace amounts of nicotine and TSNAs were observed in processed betel nut; this may be due to it being processed and packaged in the same manufacturing facility as SLT products.

According to Huque et al., the average daily use of SLT is 0.5 to 1 g SLT, 7.1x/day, which can vary by SLT type used [52]. SLT products are used extensively in South Asian countries and it is estimated that more than 90% of the global SLT users are from this region. India and Bangladesh have over 200 million SLT users [53], which is more than the rest of the world combined. Pakistan is the third largest consumer of SLT products with a prevalence among Pakistani men and women of 21 and 19%, respectively [33]. Approximately 43% of the total Bangladeshi population uses tobacco products. However, when broken down by sex and type of tobacco used, 45% of males and only 1.5% of females are smokers, while 26% of males and 28% of females are SLT users [54]. These disparate numbers indicate both a high rate of SLT use and a pronounced sex difference in the use of smoked vs. smokeless tobacco products. There are an estimated 14 million women who use SLT products in Bangladesh, and only 2.5% of them also smoke cigarettes, leaving 13.6 million Bangladeshi women who only use SLT products [31, 32]. A similar pattern is observed in India, where 33% of men and 18% of women use SLT products, with over 85% of females using SLT products exclusively [54]. By comparison, while the total SLT-using population in the USA is 8.7 million, many also smoke cigarettes [55].

The high prevalence of SLT use in South Asian countries has been correlated with the high rate of upper aerodigestive tract cancer and other tobacco-related diseases in this population. Based on data from the American Institute for Cancer Research, Bangladesh is first among countries in the incidence of hypopharyngeal cancer, third in lip and oral cavity cancer, 17^th^ in laryngeal cancer, and 18^th^ in oropharyngeal cancer [56]. In addition, Bangladesh is fourth in esophageal cancer. In 2018, the combined cancer incidence rates for Bangladeshi upper aerodigestive tract cancers exceeded 34 per 100,000 people [30], and the high TSNA levels in Bangladeshi SLT brands may be associated with the high upper aerodigestive tract cancer incidence observed in this population. Of the five most common cancers in Bangladeshi men, 19.5% were attributed to lip and oral cancers [based on 2014 data, [57]]. Despite the low smoking rates in the female Bangladeshi population, lip and oral cancers comprised 8.8% of the five most common cancers in the same year. Similarly, of the five most common cancers in Bangladeshi men, 23.4% were attributed to esophageal cancers (based on 2014 data). Despite the low smoking rates in the female Bangladeshi population, esophageal cancer comprised 13.7% of the five most common cancers in the same year. In addition, there has been an upward trend of upper aerodigestive tract cancers in Bangladeshi women. For example, among the top five most prevalent cancers, the incidence of lip and oral cancer increased by 34% in Bangladeshi women in 2018; with no increase observed in Bangladeshi men [57, 58]. Therefore, it is crucial to investigate whether this selective use of SLT products in Bangladeshi women is playing a role in the recent increases in oral cancer incidence in this population. While the data presented in the current study are suggestive of a role for SLT in aerodigestive tracts cancers in Bangladesh, future epidemiological studies will be necessary to fully evaluate this complex relationship.

## Conclusions

This study is the first to fully characterize TSNA levels in Bangladeshi smokeless tobacco (SLT) products. The high levels of TSNAs in Bangladeshi SLT brands may be an important factor contributing to the high rates of upper aerodigestive tract cancer in Bangladesh

## Supporting information

S1 Table Vendor and lot number for SLT products examined in this study. (DOCX)
