# Supplementary material for: Comparison of tobacco-specific nitrosamine levels in smokeless tobacco products: High levels in products from Bangladesh
Source: PLoS One. 2020 May 26;15(5):e0233111. doi: 10.1371/journal.pone.0233111 (PMC7250445; doi:10.1371/journal.pone.0233111)
Supplement: S1 Table — (DOCX) [file pone.0233111.s001.docx]

| Brand name | Vendor name | Lot no. | Brand name | Vendor name | Lot no. |
| --- | --- | --- | --- | --- | --- |
| Bangladesh Brands |  |  | Indian brands |  |  |
| Shohag Zarda | Samir chemical Company | 01 S | Baba Zarda | NA | NA |
| Nurani C Z | Monica chemical company | NM | Paanparag Zarda | NA | NA |
| Momo Zarda | Momo Chemical Company | 01/16 | Goa 1000 Zarda | NA | NA |
| Akiz Z | Akiz zarda factory | NM | Gopal Zarda | NA | NA |
| Chadpuri shah zarda | B. H. Chemical works | NM | Tulshi ghutka mix | NA | NA |
| Nurani Z | Al amin zarda factory | NM | Shimla Chap | NA | NA |
| Babuls Z | Babul chemical company | NM |  |  |  |
| Zakir Zarda | Zakir zarda factory | NM | Pakistani brands |  |  |
| Alo Zarda | Alo chemical company | NM | Manipuri | NA | NA |
| Halimpuri Z | NA | NM | Naswar | NA | NA |
| Gurudev Zarda | Shapan chemical works | NM |  | | |
| Dulals pati jarda | Bhai bhai zarda factory | NM | USA Brands |  |  |
| Bou Z | Ma zarda chemical co | NM | Skoal long cut | US smokeless tobacco | HCD60057I |
| Saudia Z | Panshahi zarda factroy | NM | Copenhagen snuff | NA | UHE61834N |
| Noman Z | L. Rahman Company | 7 | Kodiak premium | American snuff co | S11J4JH6 |
| Hakimpuri patti zarda | Shiohel chemical works | 982287 | Grizzly long cut | American snuff co | S1305EF7 |
| Read leaf Z | Bangla tobacco company industries | NM | Grizzly extra long | American snuff co | D21M64E7 |
| Tofan royal zarda | Jahangir chemical workshop BD | NM | Grizzly premium | American snuff co | D22J7TF7 |
| Shova Z | Junaid bissus zarda flight | NM |  |  |  |
| Ezma Shova zarda | Ezma zarda factory | 2 |  |  |  |
| Murubbi baba Z | Noor-mohammaed chemical works | NM |  |  |  |
| Aadi bhija Z | F. Rahman company | NM |  |  |  |
| Bogh gul | Mostafa gul factory | NM |  |  |  |
| Mostafa gul | Mostafa gul factory | NM |  |  |  |
| Bidut gul | Ratna chemical company | NM |  |  |  |
| Shahi eagle gul | A Quadir eagle tobacco company | NM |  |  |  |
| Shada Pata | Locally bought from Dhaka, Bangaldesh | NM |  | | |
| Shahzadi Shada pata | Shawon products ltd. | NM |  | | |
| Panparag PM | Emon zarda factory | NM |  |  |  |
| Shahi deluxe | Not mentioned on pack | NM |  |  |  |
| BD Sample SPM | Not mentioned on pack | NM |  |  |  |
| BD SampleBNW | Locally bought from Dhaka, Bangaldesh | NM |  |  |  |
| BD SampleBNR | Locally bought from Dhaka, Bangaldesh | NM |  |  |  |
| BD Sample BNC | Locally bought from Dhaka, Bangaldesh | NM |  |  |  |

**Supplementary Table 1.** Vendor and lot number for SLT products examined in this study.

NA: Not available; NM: Not mentioned on label
